# Re: One-day voiding diary in the evaluation of Lower Urinary Tract Symptoms in children

**DOI:** 10.1590/S1677-5538.IBJU.2023.0059

**Published:** 2023-04-05

**Authors:** Prasanna Ram

**Affiliations:** 1 Department of Urology All India Institute of Medical Sciences Bhubaneswar India Department of Urology, All India Institute of Medical Sciences, Sijua, Patrapada, Bhubaneswar

To the editor,

We read the recent article by Franck et al published in the International Brazilian Journal of Urology with great interest (1). In their single-center cross-sectional observational study, the authors analyzed ninety-eight children, of which 59 had primary monosymptomatic enuresis (PMNE) and 30 had overactive bladder (OAB) respectively. The authors concluded that a one day voiding diary (1dVD) is sufficient to assess these children. The authors further stated that the 1dVD has high sensitivity, and a good correlation to the three-day voiding diary (3dVD) when evaluating these children. The study used the maximum voided volume (MVV) as a surrogate to evaluate the bladder capacity in these children and noted that it was as close as 68% of that obtained by the expected bladder capacity (EBC).

PMNE and OAB are extremely troublesome issues in the paediatric populations, and accurate evaluation of these symptoms is crucial for effective management.

The author’s revealed that those with high post-voided residual on ultrasound or an interrupted or staccato curve on uroflowmetry were excluded in the study. A note on the reason for exclusion may add strength to the study as a child with a neurogenic bladder may also present with such symptoms and patterns, and the bladder diary is the first step in assessing these children.

As several studies in the recent past have pointed towards the superiority of a three-day voiding diary, adding a comment of the relation between a 1dVD and invasive testing would provide valuable insight on its use. A study by Lee et al. found that a three-day voiding diary was more reliable in diagnosing bladder dysfunction compared to a one-day diary (3). Another study noted that a three-day voiding diary had a higher diagnostic accuracy in children with voiding symptoms (4).

We propose evaluating children with other forms of LUTS with a 1dVD and comparing them to a 3dVD as this can standardize the use of a 1dVD in the paediatric population.

Lastly, we would also like to congratulate the authors for their endeavors. This study could provide the pathway for future research.

The Author
